# A vast resource of allelic expression data spanning human tissues

**DOI:** 10.1186/s13059-020-02122-z

**Published:** 2020-09-11

**Authors:** Stephane E. Castel, François Aguet, Pejman Mohammadi, GTEx Consortium, François Aguet, Shankara Anand, Kristin G. Ardlie, Stacey Gabriel, Gad A. Getz, Aaron Graubert, Kane Hadley, Robert E. Handsaker, Katherine H. Huang, Seva Kashin, Xiao Li, Daniel G. MacArthur, Samuel R. Meier, Jared L. Nedzel, Duyen T. Nguyen, Ayellet V. Segrè, Ellen Todres, François Aguet, Shankara Anand, Kristin G. Ardlie, Brunilda Balliu, Alvaro N. Barbeira, Alexis Battle, Rodrigo Bonazzola, Andrew Brown, Christopher D. Brown, Stephane E. Castel, Donald F. Conrad, Daniel J. Cotter, Nancy Cox, Sayantan Das, Olivia M. de Goede, Emmanouil T. Dermitzakis, Jonah Einson, Barbara E. Engelhardt, Eleazar Eskin, Tiffany Y. Eulalio, Nicole M. Ferraro, Elise D. Flynn, Laure Fresard, Eric R. Gamazon, Diego Garrido-Martín, Nicole R. Gay, Gad A. Getz, Michael J. Gloudemans, Aaron Graubert, Roderic Guigó, Kane Hadley, Andrew R. Hame, Robert E. Handsaker, Yuan He, Paul J. Hoffman, Farhad Hormozdiari, Lei Hou, Katherine H. Huang, Hae Kyung Im, Brian Jo, Silva Kasela, Seva Kashin, Manolis Kellis, Sarah Kim-Hellmuth, Alan Kwong, Tuuli Lappalainen, Xiao Li, Xin Li, Yanyu Liang, Daniel G. MacArthur, Serghei Mangul, Samuel R. Meier, Pejman Mohammadi, Stephen B. Montgomery, Manuel Muñoz-Aguirre, Daniel C. Nachun, Jared L. Nedzel, Duyen T. Nguyen, Andrew B. Nobel, Meritxell Oliva, Yo Son Park, Yongjin Park, Princy Parsana, Abhiram S. Rao, Ferran Reverter, John M. Rouhana, Chiara Sabatti, Ashis Saha, Ayellet V. Segrè, Andrew D. Skol, Matthew Stephens, Barbara E. Stranger, Benjamin J. Strober, Nicole A. Teran, Ellen Todres, Ana Viñuela, Gao Wang, Xiaoquan Wen, Fred Wright, Valentin Wucher, Yuxin Zou, Pedro G. Ferreira, Gen Li, Marta Melé, Esti Yeger-Lotem, Mary E. Barcus, Debra Bradbury, Tanya Krubit, Jeffrey A. McLean, Liqun Qi, Karna Robinson, Nancy V. Roche, Anna M. Smith, Leslie Sobin, David E. Tabor, Anita Undale, Jason Bridge, Lori E. Brigham, Barbara A. Foster, Bryan M. Gillard, Richard Hasz, Marcus Hunter, Christopher Johns, Mark Johnson, Ellen Karasik, Gene Kopen, William F. Leinweber, Alisa McDonald, Michael T. Moser, Kevin Myer, Kimberley D. Ramsey, Brian Roe, Saboor Shad, Jeffrey A. Thomas, Gary Walters, Michael Washington, Joseph Wheeler, Scott D. Jewell, Daniel C. Rohrer, Dana R. Valley, David A. Davis, Deborah C. Mash, Mary E. Barcus, Philip A. Branton, Leslie Sobin, Laura K. Barker, Heather M. Gardiner, Maghboeba Mosavel, Laura A. Siminoff, Paul Flicek, Maximilian Haeussler, Thomas Juettemann, W. James Kent, Christopher M. Lee, Conner C. Powell, Kate R. Rosenbloom, Magali Ruffier, Dan Sheppard, Kieron Taylor, Stephen J. Trevanion, Daniel R. Zerbino, Nathan S. Abell, Joshua Akey, Lin Chen, Kathryn Demanelis, Jennifer A. Doherty, Andrew P. Feinberg, Kasper D. Hansen, Peter F. Hickey, Lei Hou, Farzana Jasmine, Lihua Jiang, Rajinder Kaul, Manolis Kellis, Muhammad G. Kibriya, Jin Billy Li, Qin Li, Shin Lin, Sandra E. Linder, Stephen B. Montgomery, Meritxell Oliva, Yongjin Park, Brandon L. Pierce, Lindsay F. Rizzardi, Andrew D. Skol, Kevin S. Smith, Michael Snyder, John Stamatoyannopoulos, Barbara E. Stranger, Hua Tang, Meng Wang, Philip A. Branton, Latarsha J. Carithers, Ping Guan, Susan E. Koester, A. Roger Little, Helen M. Moore, Concepcion R. Nierras, Abhi K. Rao, Jimmie B. Vaught, Simona Volpi, Kristin G. Ardlie, Tuuli Lappalainen

**Affiliations:** 1 New York Genome Center, New York, NY USA; 2 Department of Systems Biology, Columbia University, New York, NY USA; 3 The Broad Institute of MIT and Harvard, Cambridge, USA; 4 Department of Integrative Structural and Computational Biology, The Scripps Research Institute, La Jolla, CA USA; 5 Scripps Translational Science Institute, La Jolla, CA USA

**Keywords:** ASE, Allelic expression, eQTL, Regulatory variation, Genomics, Functional genomics, GTEx

## Abstract

Allele expression (AE) analysis robustly measures *cis*-regulatory effects. Here, we present and demonstrate the utility of a vast AE resource generated from the GTEx v8 release, containing 15,253 samples spanning 54 human tissues for a total of 431 million measurements of AE at the SNP level and 153 million measurements at the haplotype level. In addition, we develop an extension of our tool phASER that allows effect sizes of *cis*-regulatory variants to be estimated using haplotype-level AE data. This AE resource is the largest to date, and we are able to make haplotype-level data publicly available. We anticipate that the availability of this resource will enable future studies of regulatory variation across human tissues.

## Background

**Fig. 1:**
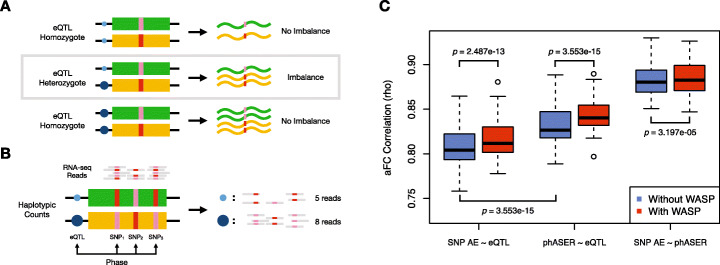
Capturing *cis*-regulatory effects with phased allelic expression data. **a** The presence of a heterozygous *cis-*regulatory variant or eQTL produces an expression-level imbalance between the two haplotypes, which can be detected using allelic expression analysis. **b** RNA-seq reads overlapping heterozygous SNPs in expressed regions of the gene can be used to quantify the expression of alleles relative to one another. These SNPs can be phased with each other and their counts aggregated to produce haplotype-level expression estimates, or haplotypic counts. The effects of regulatory variants can be captured by phasing them with haplotypic counts. **c** Spearman correlation across the 49 GTEx v8 tissues where eQTLs were called between eQTL effect size (allelic fold change, aFC) and effect size measured using AE data from the single SNP with the highest coverage (SNP AE) or haplotype-level AE generated with phASER (phASER). Results are shown with and without allelic mapping bias correction from WASP. In each tissue, only a single top significant (FDR < 5%) eQTL per gene was analyzed. *p* values were calculated using a Wilcoxon paired signed rank test. For boxplots, bottom whisker: Q1 − 1.5*interquartile range (IQR), top whisker: Q3 + 1.5*IQR, box: IQR, and center: median

Allelic expression (AE, also known as allele-specific expression or ASE) analysis is a powerful technique that can be used to measure the expression of gene alleles relative to one another within single individuals. This makes it well suited to measure *cis*-acting regulatory variation using imbalance between alleles in heterozygous individuals (Fig. 1a) [1]. AE analysis can capture both common *cis*-regulatory variation, for example, expression quantitative trait loci (eQTLs), and rare regulatory variation [2]. It can also be used to measure allele-specific epigenetic effects such as parent of origin imprinting [3].

In practice, AE analysis uses RNA-seq reads that overlap heterozygous single nucleotide polymorphisms (SNPs), where the SNP can be used to assign the read to an allele. These heterozygous SNPs capture the cumulative effects of *cis*-regulatory variation acting on each allele. Allelic imbalance occurs when the two alleles of a gene are expressed at different levels. The magnitude of the imbalance can be quantified by allelic fold change (aFC) [1], and the statistical significance of the imbalance can be evaluated using binomial-based statistics to account for the count-based nature of the data [4]. In some cases, these effects can be caused by the SNPs being used to measure AE themselves, for example, stop-gain variants that cause nonsense-mediated decay (NMD) [5], but often they simply capture the effects of other *cis*-acting variation. Traditionally, a single SNP has been used to measure AE, by taking the SNP with the highest coverage per gene. However, as a result of improvements in genome phasing, data can be aggregated across SNPs to produce estimates of AE at the haplotype level (Fig. 1b). We have previously developed a tool, phASER, which does this systematically, in a way that uses the information contained within reads to improve phasing, while preventing double counting of reads across SNPs to improve the quality of data generated [6].

In this work, we present and demonstrate the utility of an AE resource generated using the Genotype Tissue Expression (GTEx) version 8 data release comprising RNA-seq data from 54 tissues and 838 individuals, for a total of 15,253 samples [7]. We generated both SNP-level and haplotype-level AE data. While the SNP-level data is available to approved users through dbGaP, the haplotype-level data does not contain identifiable information, and we were thus able to make it publicly available on the GTEx portal. Finally, we developed an addition to phASER, called phASER-POP which makes it easy to generate population-scale, haplotype-level AE data and calculate effect sizes for regulatory variants.

## Results and discussion

Both SNP-level and haplotype-level AE data were generated for each GTEx sample using current best practices, both with and without using WASP filtering [8] to reduce the mapping bias that is sometimes present in AE analysis, resulting in 4 data types per sample (Additional file 1: Fig. S1, “Data generation and availability” section in the “Methods” section). Across samples, this produced over 431 million measurements of AE at the SNP level and 153 million measurements of AE at the haplotype level. To demonstrate the ability of these data to robustly capture *cis*-regulatory effects and also benchmark the four data types relative to one another, we estimated eQTL effect sizes across the 49 tissues where eQTLs were mapped from AE data using allelic fold change (aFC) and compared them to those derived from eQTL mapping [7]. The effect sizes were quantified using aFC for both AE and eQTL data. To make it easier to generate aFC estimates for regulatory variants from phASER data, we developed a new add-on to the software package, phASER-POP, eliminating the need for custom scripts (Additional file 1: Fig. S2). Briefly, phASER-POP integrates genotype calls and haplotype-level AE data across individuals and phases each regulatory variant of interest (e.g., eQTL) in each individual with their AE data. It then calculates statistics, including aFC per sample, and its median across samples for individuals that are heterozygous for the variant. At the sample level, aFC is a net expression fold difference between the two haplotypes in an individual that is affected by all heterozygous regulatory variants, including other eQTLs and rare regulatory variation, and thus can differ from the expected aFC derived from eQTL mapping. However, the median aFC across all individuals in a population that is heterozygous for a given eQTL can be used as a robust estimate of its effect size [1]. The software is described in full detail in the “Methods” section.

To characterize the GTEx AE resource, we first compared aFC estimates calculated for GTEx eQTLs between SNP- and haplotype-level AE data. We found high correlations between AE and eQTL estimates, with a median Spearman rho of 0.80 across tissues for SNP-level data and 0.83 for haplotype-level data generated by phASER (Fig. 1c). Haplotype-level correlations were significantly higher than SNP-level correlations (*p* = 3.55e−15, Wilcoxon paired signed rank test) while at the same time producing estimates for a median of 20% more eQTLs (Additional file 1: Fig. S3). Based on this, we recommend using the haplotype-level data for most downstream analyses, as it yields more data of a higher quality. However, there are some circumstances when the SNP-level data should be used. For example, when analyzing allelic splicing, the haplotype-level data is not appropriate because it spans the entire transcript, whereas only SNPs within the exon(s) or intron(s) of interest should be analyzed. Furthermore, when analyzing transcribed variants with post-transcriptional effects on gene expression, such as stop-gain or splice variants, SNP-level AE data from the variant of interest is more straightforward to analyze.

Next, we assessed the effect of read mapping bias correction on allelic expression analysis by comparing eQTL and AE effect size correlations with and without WASP filtering. WASP filtering significantly improved correlations for both SNP- (*p* = 2.49e−13, median improvement 1.22%) and haplotype- (*p* = 3.55e−15, median improvement 1.28%) level data (Fig. 1c). Since WASP works by removing, rather than correcting reads with mapping bias, we compared the number of eQTLs for which an aFC estimate could be calculated and found only a small 3.5% reduction (Additional file 1: Fig. S3d). We therefore recommend using WASP-filtered data for most downstream analyses. This is particularly important if the aim is to identify strong signals of allelic imbalance, which can often be false positives due to mapping bias. We encourage users of the resource to assess the impact of WASP filtering for their own use case, so have included the unfiltered AE data for comparison.

**Fig. 2:**
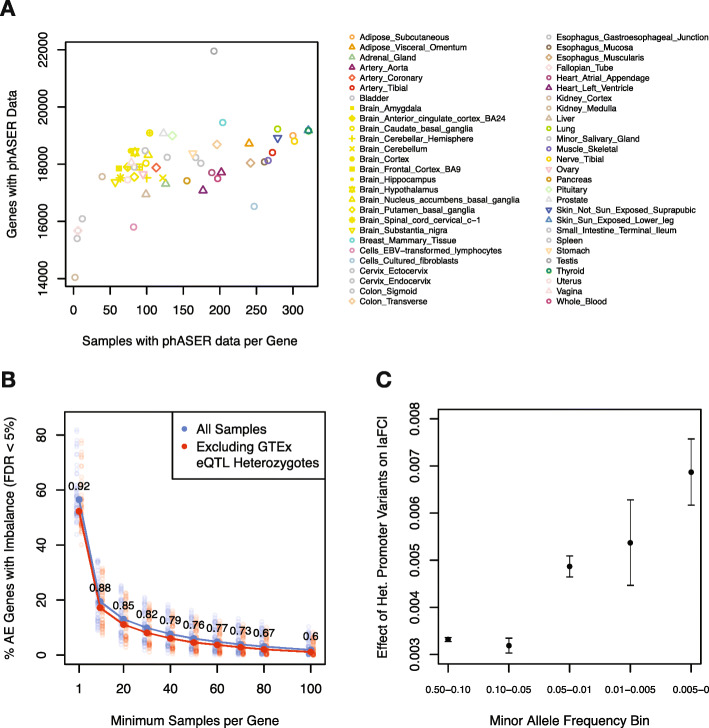
The GTEx v8 haplotype-level allelic expression resource. **a** Number of genes per tissue with haplotype-level AE data (AE genes) in at least 1 individual versus the median number of samples with data per gene. **b** Percentage of AE genes with significant allelic imbalance (binomial test, gene-level FDR < 5%) in at least *n* samples per gene using all samples (blue) or excluding samples heterozygous for any top (FDR < 5%) or independent GTEx eQTL (permutation *p* < 1e−4) (red). Faded points are values for individual tissues, and solid points are the median across tissues. Proportions above data points indicate the reduction in percentage of AE genes with imbalance after removing eQTL heterozygotes. A full summary of these statistics across tissues and sample thresholds is available in Additional file 3: Table S2. **c** The effect of the number of heterozygous variants in or proximal to gene promoters (< 10 kb upstream of TSS) on allelic imbalance stratified by minor allele frequency. Plotted values are effect estimates and 95% confidence intervals (see the “Promoter variant effect modeling” section in the “Methods” section)

Next, we characterized the WASP-filtered AE data. In the GTEx RNA-seq data, at a minimum coverage of 8 reads, samples had a median of 7,607 genes with AE data at the SNP level and 10,043 genes at the haplotype level, and this dropped as a function of increasing coverage thresholds (Additional file 1: Fig. S4). With the same coverage threshold, at the tissue level and excluding tissues with small sample sizes (*N* < 70) where eQTL mapping was not performed, there were a median of 18,042 genes with a median of 128 samples per gene using haplotype-level AE data, rendering the data set well-powered to detect *cis*-regulatory effects (Fig. 2a). The median number of samples with AE data per gene was largely dependent on tissue sample size, ranging from 39 for kidney cortex (*N* = 73 samples) to 321 for thyroid (*N* = 574 samples). The number of genes with AE data was correlated with both sample size (rho = 0.41) and the number of expressed genes (rho = 0.82), with the two cell lines having the lowest number of genes with AE data (LCLs = 15,804, fibroblasts = 16,526) and the testis having the largest number of genes with AE data (21,952) despite an intermediate sample size of 322 (Additional file 2: Table S1). This was likely driven by the number of expressed genes in testes, which was the highest across all tissues.

Finally, we sought to demonstrate the pervasiveness of *cis*-regulatory effects that can be captured with this resource. We found that even strong regulatory effects, where one allele was expressed at ≥ 2x the level of the other allele, are widely present, even for protein-coding genes, with 53% of protein-coding genes showing such an effect in at least one tissue and at least 50 individuals (Additional file 1: Fig. S5). Considering all genes, we found that a median of 10,183 genes (or a median of 56% of those genes with AE data) per tissue exhibited significant allelic imbalance (binomial test, FDR < 5% at the gene level) in at least one sample, indicating the wide-spread nature of *cis*-regulatory effects (Fig. 2b). Removing individuals that were heterozygous for any known GTEx eQTL (“GTEx eQTLs” section in the “Methods” section) only resulted in a median reduction of 7.5% in the number of genes with significant imbalance in at least one sample, demonstrating the potential of this resource to identify additional regulatory effects, including rare regulatory effects, that are not captured in eQTL analysis. To further demonstrate this potential, we modeled allelic imbalance as a function of the minor allele frequency and number of heterozygous variants found in or proximal to gene promoters (< 10 kb upstream of TSS). As expected, we found that rare variants tended to have larger effects on allelic imbalance than common variants, with the rarest class of variants analyzed (MAF < 0.005 in GTEx) having the strongest effects (Fig. 2c).

## Conclusion

In this work, we used the GTEx v8 release to produce a vast allelic expression resource, consisting of hundreds of millions of measurements. We generated SNP- and haplotype-level data, which provides better estimates of allelic expression for a greater number of genes. These data have numerous uses for the study of regulatory variation. SNP-level data from the previous v6 AE dataset [2] has been extensively used to study gene regulation, for example, to study the effects of rare regulatory variation [9], X chromosome inactivation [10], Neanderthal-introgressed regulatory variation [11], interaction between regulatory and coding variants [12], and regulatory constraint in the context of rare disease [13]. The haplotype-level v8 data presented here have similarly found broad use for studying gene regulation. For example, they have been used to replicate sex-, population-, and cell type-specific eQTLs [7, 14] as well as capture the effects of rare regulatory variants [15] and study *cis*-domains of lncRNA regulation [16]. By making haplotype-level AE data publicly available for the first time, we anticipate that this resource will find similarly broad use as the eQTL data it complements.

## Methods

### Data generation and availability

Paired-end 75-bp Illumina RNA-seq reads were aligned to hg38 using STAR [17] v2.5.3a (without allelic mapping bias correction) and v2.6.0c (with allelic mapping bias correction) in two-pass mode, and with allelic mapping bias correction enabled via the --waspOutputMode option which replicates the approach in van de Geijn et al. [8] (the full settings of the alignment pipeline are described at https://github.com/broadinstitute/gtex-pipeline). All data was generated with or without using this feature and is indicated by “_WASP_” in the file names.

SNP-level AE data was generated using the GATK ASEReadCounter tool v3.8-0-ge9d806836 with the following settings: -U ALLOW_N_CIGAR_READS -minDepth 1 --allow_potentially_misencoded_quality_scores --minMappingQuality 255 --minBaseQuality 10. Raw SNP-level data, consisting of the GATK tool output, were aggregated per subject across all tissues. Raw autosomal SNP-level data, for SNPs with ≥ 8 reads, was annotated by assigning heterozygous SNPs to genes using Gencode v26, calculating the expected null ratio for each combination of ref/alt allele [4], calculating a binomial *p* value by comparing to the expected null ratio, calculating a multiple hypothesis corrected *p* value per tissue using Benjamini-Hochberg, and flagging sites that overlapped low-mappability regions (75-mer mappability < 1 based on 75mer alignments with up to two mismatches based on the pipeline for ENCODE tracks and available on the GTEx portal), showed mapping bias in simulation [18], or had no more reads supporting two alleles than would be expected from sequencing noise alone, indicating potential genotyping errors (FDR < 1%, see Castel et al. [4] for the description of the test). The genotype warning test cannot distinguish between strong allelic expression and a true genotyping error and as a result should not be used when studying phenomena with expected mono-allelic expression (e.g., imprinting).

Haplotype-level data was generated using phASER v1.0.1 [6]. phASER was run using whole genome sequencing genotype calls that were population-phased with Shapeit v2.837 in read-backed phasing mode with whole genome sequencing reads [19]. phASER was run using all available RNA-seq libraries per subject. RNA-seq read-backed phased genotype data are provided (filename: phASER_GTEx_v8_merged.vcf.gz). Haplotypic expression was calculated using phASER Gene AE 1.2.0 and Gencode v26 gene annotations with min_haplo_maf 0.01. Haplotypic expression matrices containing all samples were generated using the “phaser_expr_matrix.py” script. This consists of a single string per sample per gene with the format “HAP_A_COUNT|HAP_B_COUNT.” One matrix was generated using only haplotypes that could be genome-wide phased such that the haplotype assignment is consistent across genes within an individual and with the phased VCF (filename: phASER_GTEx_v8_matrix.gw_phased.txt.gz). Another was generated that does not ensure genome-wide haplotype phasing across genes, which includes more counts, but makes the haplotype assignment of A/B arbitrary and unrelated across genes within an individual or the VCF (filename: phASER_GTEx_v8_matrix.txt.gz). The full settings of the haplotype-level AE pipeline are described at https://github.com/broadinstitute/gtex-pipeline/.

SNP-level data is available for authorized users via dbGaP under accession phs000424 (filenames: phe000039.v1.GTEx_v8_ASE.expression-matrixfmt-ase.c1.GRU.tar, phe000039.v1.GTEx_v8_ASE_WASP.expression-matrixfmt-ase.c1.GRU.tar) [20]. phASER-generated, haplotype-level data is available through the same dbGaP accession (folders GTEx_Analysis_v8_phASER and GTEx_Analysis_v8_phASER_WASP inside archive phe000037.v1.GTEx_v8_RNAseq.expression-data-matrixfmt.c1.GRU.tar) and on the GTEx Portal (http://gtexportal.org/).

Unless stated otherwise, all analyses were performed using only protein-coding and lncRNA genes.

### Software and availability

The original phASER package produced gene-level haplotypic expression per individual [6]. We developed new additions to phASER (phASER-POP) that make it easier to analyze data across many samples, as is often done with gene expression quantifications. First, we developed a new addition to the software (phaser_expr_matrix.py) that enables the aggregation of gene-level haplotypic expression measurement files across samples to produce a single haplotypic expression matrix, where each row is a gene and each column is a sample. The values consist of a single string per sample per gene in the format “HAP_A_COUNT|HAP_B_COUNT.” This format is intended to facilitate downstream analyses of allelic expression.

Second, we developed a tool to make it easier to estimate effect sizes of regulatory variants using phASER haplotypic expression data (phaser_cis_var.py). As input, this script takes a phASER haplotype expression matrix, a phased VCF, and a list of regulatory variants (e.g., eQTLs) to calculate effect sizes for. To improve accuracy, the read-backed phased VCFs produced by phASER should be used, but first need to be combined across individuals, which can be performed using, e.g., “bcftools merge ind1.vcf.gz ind2.vcf.gz ….” Using these inputs, the tool phases each regulatory variant of interest with haplotype-level expression data in each individual. It then calculates numerous statistics, including allelic fold change (aFC) [1] per sample, and a median across samples for individuals that are heterozygous for the variant of interest. This median can be used as an estimate of regulatory variant effect size. aFC is calculated as log_2_((eqtl_alt_allele_haplotype_count+1)/(eqtl_ref_allele_haplotype_count+1)). The output also includes aFC estimates calculated for homozygous individuals and performs a ranksum test of absolute aFC in heterozygotes as compared to homozygotes. True regulatory variants are expected to have a significantly higher aFC in heterozygous individuals. 95% confidence intervals are included for all aFC estimates, and all underlying individual data, including haplotypic counts, are outputted.

The updated phASER package code along with extensive documentation is available through GitHub at https://github.com/secastel/phaser/tree/master/phaser_pop under the GNU General Public License v3 [21].

### GTEx eQTLs

For comparison between eQTL effect size and allelic expression effect size, GTEx v8 top significant (FDR < 5%) eQTLs were used from 49 tissues [7]. This results in at most a single eQTL per gene in a given tissue. When quantifying the number of samples that are not heterozygous for a known eQTL but still show allelic imbalance, gene-level haplotypic expression levels were excluded for a sample if the individual was heterozygous for a top significant eQTL or a nominally significant (permutation *p* < 1e−4) independent eQTL in any of the 49 tissues.

### Promoter variant effect modeling

The effects of regulatory variants in or proximal to gene promoters were modeled using haplotype-level allelic expression data. Briefly, for each individual, all heterozygous variants within 10 kb upstream of protein-coding or lincRNA gene transcription start sites (TSS) were retrieved and the median allelic imbalance for that gene across all tissues, measured using aFC, was calculated. For each individual by gene, the number of heterozygous variants (which could potentially cause allelic imbalance) falling into each of the following minor allele frequency (MAF) bins was calculated: 0.50–0.10, 0.10–0.05, 0.05–0.01, 0.01–0.005, 0.005–0. Bins were inclusive of variants whose MAF < upper bin limit and ≥ the lower bin limit. Using data from all genes by individuals, absolute aFC was modeled with a multivariate linear model (speedglm function in R) using the number of variants in each of the MAF bins as predictors. The coefficients for each of the predictors were then plotted along with their 95% confidence intervals (confint function in R) as a measure of the effect of the number of heterozygous variants in each MAF class on allelic imbalance, with a higher coefficient indicating a stronger effect (i.e., a larger allelic imbalance). Because allele frequencies were calculated within the GTEx cohort, only individuals of predominantly European ancestry (*N* = 699, determined by PCA) were included in the analysis, to ensure accurate allele frequency estimates. Without this filtering, population-specific variants, whose populations are not well represented in the GTEx cohort, may have inaccurate, likely underestimated allele frequencies, which can confound the analysis.

## Supplementary information

**Additional file 1: **Supplemental **Figures S1-S5**.

**Additional file 2: Table S1**: Tissue-level summary statistics for haplotype-level AE data. Table listing sample size, number of expressed genes (defined as genes with > = 0.1 TPM in at least 1 individual), number of genes with phASER data (defined as genes with > = 8 reads in at least 1 individual), median number of samples per gene with phASER data, and if the tissue was used for GTEx v8 eQTL mapping.

**Additional file 3: Table S2**: Sample-threshold and allelic imbalance statistics for haplotype-level AE data. Table where rows are each of the 49 GTEx tissues where eQTLs were called and columns list the number of genes with haplotype-level AE data at minimum number of sample thresholds from 1 to 300 (minXXX). For example, min1 lists the number of genes that have AE data from at least 1 sample. The table has three sheets, the first (all_data) presents statistics generated using all haplotype-level AE data, the second (sig_imb_fdr05), counting only cases with significant allelic imbalance (binomial test versus 50/50, gene-level FDR < 5%), and finally (sig_imb_fdr05_no_het), counting only cases with significant imbalance where the individual is not heterozygous for any top (FDR < 5%) or independent (permutation *p* < 1e-4) eQTLs across any GTEx tissues for the gene.

**Additional file 4.** Review history.

## Data Availability

The datasets generated and/or analyzed during the current study are available to authorized users via dbGaP under accession phs000424 [20] and on the GTEx portal (http://gtexportal.org/). The updated phASER package code along with extensive documentation is available through GitHub at https://github.com/secastel/phaser/tree/master/phaser_pop under the GNU General Public License v3 [21].

## References

[1] Mohammadi P, Castel S, Brown A, Lappalainen T (2017). Quantifying the regulatory effect size of cis-acting genetic variation using allelic fold change. Genome Res.

[2] GTEx Consortium (2017). Genetic effects on gene expression across human tissues. Nature.

[3] Baran, Y., Subramaniam, M., Biton, A., Tukiainen, T., Tsang, E., Rivas, M., Pirinen, M., Gutierrez-Arcelus, M., Smith, K., Kukurba, K., Zhang, R., Eng, C., Torgerson, D., Urbanek, C., Consortium, t., Li, J., Rodriguez-Santana, J., Burchard, E., Seibold, M., MacArthur, D., Montgomery, S., Zaitlen, N., Lappalainen, T. (2015). The landscape of genomic imprinting across diverse adult human tissues. Genome Res 25(7), 927–936. 10.1101/gr.192278.115 PMC4484390 25953952

[4] Castel S, Levy-Moonshine A, Mohammadi P, Banks E, Lappalainen T (2015). Tools and best practices for data processing in allelic expression analysis. Genome Biol.

[5] Rivas M, Pirinen M, Conrad D, Lek M, Tsang E, Karczewski K, Maller J, Kukurba K, DeLuca D, Fromer M, Ferreira P, Smith K, Zhang R, Zhao F, Banks E, Poplin R, Ruderfer D, Purcell S, Tukiainen T, Minikel E, Stenson P, Cooper D, Huang K, Sullivan T, Nedzel J, Consortium T, Consortium T, Bustamante C, Li J, Daly M, Guigo R, Donnelly P, Ardlie K, Sammeth M, Dermitzakis E, McCarthy M, Montgomery S, Lappalainen T, MacArthur D (2015). Effect of predicted protein-truncating genetic variants on the human transcriptome. Science.

[6] Castel S, Mohammadi P, Chung W, Shen Y, Lappalainen T (2016). Rare variant phasing and haplotypic expression from RNA sequencing with phASER. Nat Commun.

[7] GTEx Consortium (2019). The GTEx Consortium atlas of genetic regulatory effects across human tissues.

[8] Geijn B, McVicker G, Gilad Y, Pritchard J (2015). WASP: allele-specific software for robust molecular quantitative trait locus discovery. Nat Methods.

[9] Li X, Kim Y, Tsang EK, Davis JR, Damani FN, Chiang C (2017). The impact of rare variation on gene expression across tissues. Nature.

[10] Tukiainen T, Villani AC, Yen A, Rivas MA, Marshall JL, Satija R (2017). Landscape of X chromosome inactivation across human tissues. Nature.

[11] McCoy RC, Wakefield J, Akey JM (2017). Impacts of Neanderthal-introgressed sequences on the landscape of human gene expression. Cell.

[12] Castel SE, Cervera A, Mohammadi P, Aguet F, Reverter F, Wolman A (2018). Modified penetrance of coding variants by cis-regulatory variation contributes to disease risk. Nat Genet.

[13] Mohammadi P, Castel SE, Cummings BB, Einson J, Sousa C, Hoffman P (2019). Genetic regulatory variation in populations informs transcriptome analysis in rare disease. Science.

[14] S. Kim-Hellmuth*, F. Aguet*, M. Oliva*, M. Muñoz-Aguirre, V. Wucher, S. Kasela, S.E. Castel, A.R. Hamel, A. Viñuela, A.L. Roberts, S. Mangul, X. Wen, G. Wang, A.N. Barbeira, D. Garrido-Martín, B. Nadel, Y. Zou, R. Bonazzola, J. Quan, A. Brown, A. Martinez-Perez, J.M. Soria, GTEx Consortium, G. Getz, E.T. Dermitzakis, K.S. Small, M. Stephens, H.S. Xi, H.K. Im, R. Guigó, A.V. Segrè, B.E. Stranger, K.G. Ardlie, T. Lappalainen, "Cell type specific genetic regulation of gene expression across human tissues," bioRxiv, 2019. 10.1101/806117.

[15] N.M. Ferraro, B.J. Strober, J. Einson, X. Li, F. Aguet, A.N. Barbeira, S.E. Castel, J.R. Davis, A.T. Hilliard, B. Kotis, Y. Park, A.J. Scott, C. Smail, E.K. Tsang, K.G. Ardlie, T.L. Assimes, I. Hall, H.K. Im, GTEx Consortium, T. Lappalainen, P. Mohammadi, S.B. Montgomery, A. Battle, "Diverse transcriptomic signatures across human tissues identify functional rare genetic variation," bioRxiv, 2019. 10.1101/786053.

[16] O.M. de Goede, N.M. Ferraro, D.C. Nachun, A.S. Rao, F. Aguet, A.N. Barbeira, S.E. Castel, S. Kim-Hellmuth, Y. Park, A.J. Scott, B.J. Strober, GTEx Consortium, C.D. Brown, X. Wen, I. M. Hall, A. Battle, T. Lappalainen, H.K. Im, K.G. Ardlie, T. Quertermous, K. Kirkegaard, S.B. Montgomery, "Long non-coding RNA gene regulation and trait associations across human tissues," bioRxiv, 2019. 10.1101/793091.

[17] Dobin A, Davis C, Schlesinger F, Drenkow J, Zaleski C, Jha S, Batut P, Chaisson M, Gingeras T (2013). STAR: ultrafast universal RNA-seq aligner. Bioinformatics.

[18] Panousis N, Gutierrez-Arcelus M, Dermitzakis E, Lappalainen T (2014). Allelic mapping bias in RNA-sequencing is not a major confounder in eQTL studies. Genome Biol.

[19] Delaneau O, Howie B, Cox A, Zagury J-F, Marchini J (2013). Haplotype estimation using sequence reads. Am J Hum Genet.

[20] Common Fund. Genotype-Tissue Expression Project (GTEx). Database of Genotypes and Phenotypes (dbGaP). phs000424. (2019).

[21] Castel, S.E., Phasing and Allele Specific Expression from RNA-seq. Github. https://github.com/secastel/phaser. (2019).

