# Supplementary material for: A vast resource of allelic expression data spanning human tissues
Source: Genome Biol. 2020 Sep 11;21:234. doi: 10.1186/s13059-020-02122-z (PMC7488534; doi:10.1186/s13059-020-02122-z)
Supplement: Supplementary file 1 — Additional file 1: Supplemental Figures S1-S5. [file 13059_2020_2122_MOESM1_ESM.docx]

**Supplemental Figures**

**
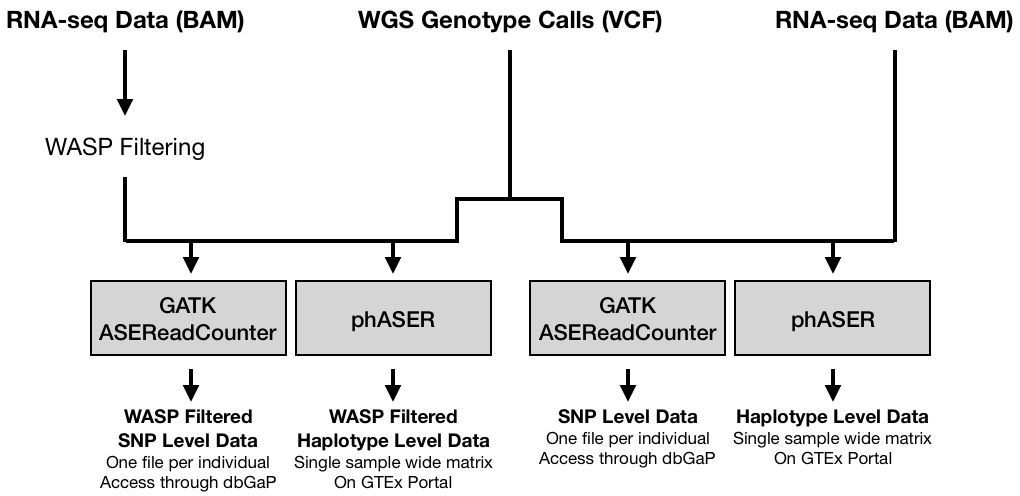
**

**Figure S1. GTEx v8 AE data types.** AE data is available at both the SNP and haplotype-level (single measurement per gene) with and without WASP filtering to reduce mapping bias. For SNP-level data, a single file was generated per individual containing data from across all tissues that were sampled from that individual and is available through dbGaP. At the haplotype-level, a matrix containing a single AE measurement per gene across all GTEx samples is available publicly through the GTEx portal.

**
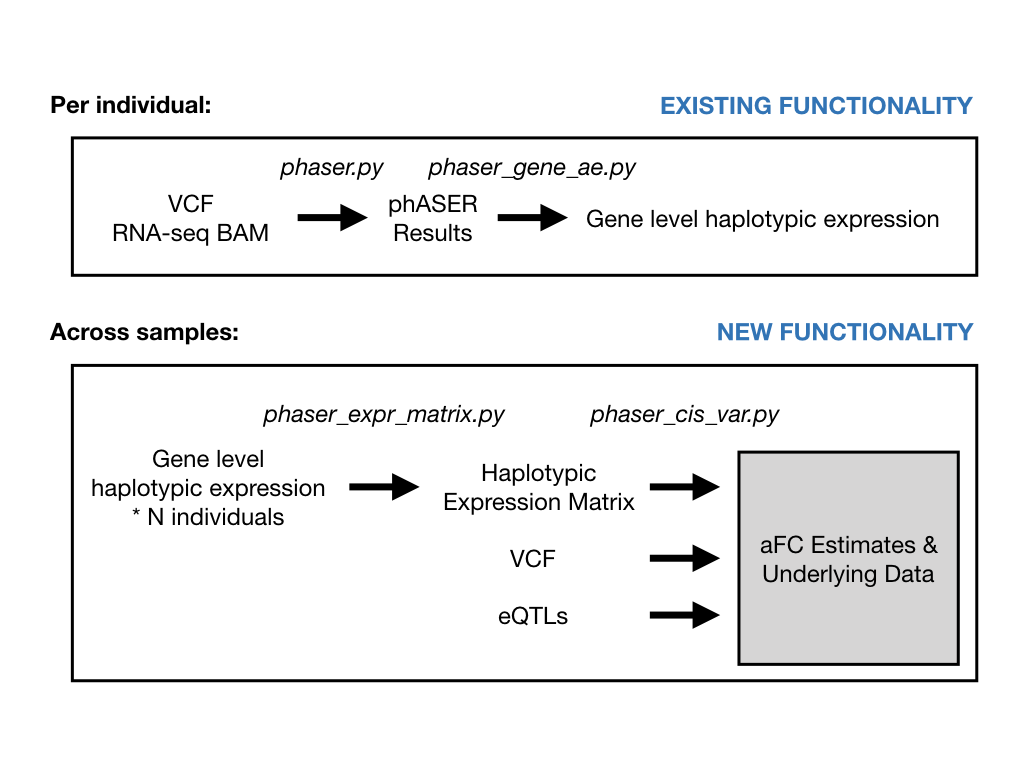
**

**Figure S2. Additions to the phASER package that allow for easy measurement of regulatory variant effects using phased AE data.** The original phASER package produced gene-level haplotypic expression per individual. The new additions allow gene-level haplotypic expression measurement files to be combined across individuals to produce a single haplotypic expression matrix, where each row is a gene and each column is an individual. This matrix can then be used to retrieve AE data and calculate relevant statistics for a given set of eQTLs or any other variants of interest.


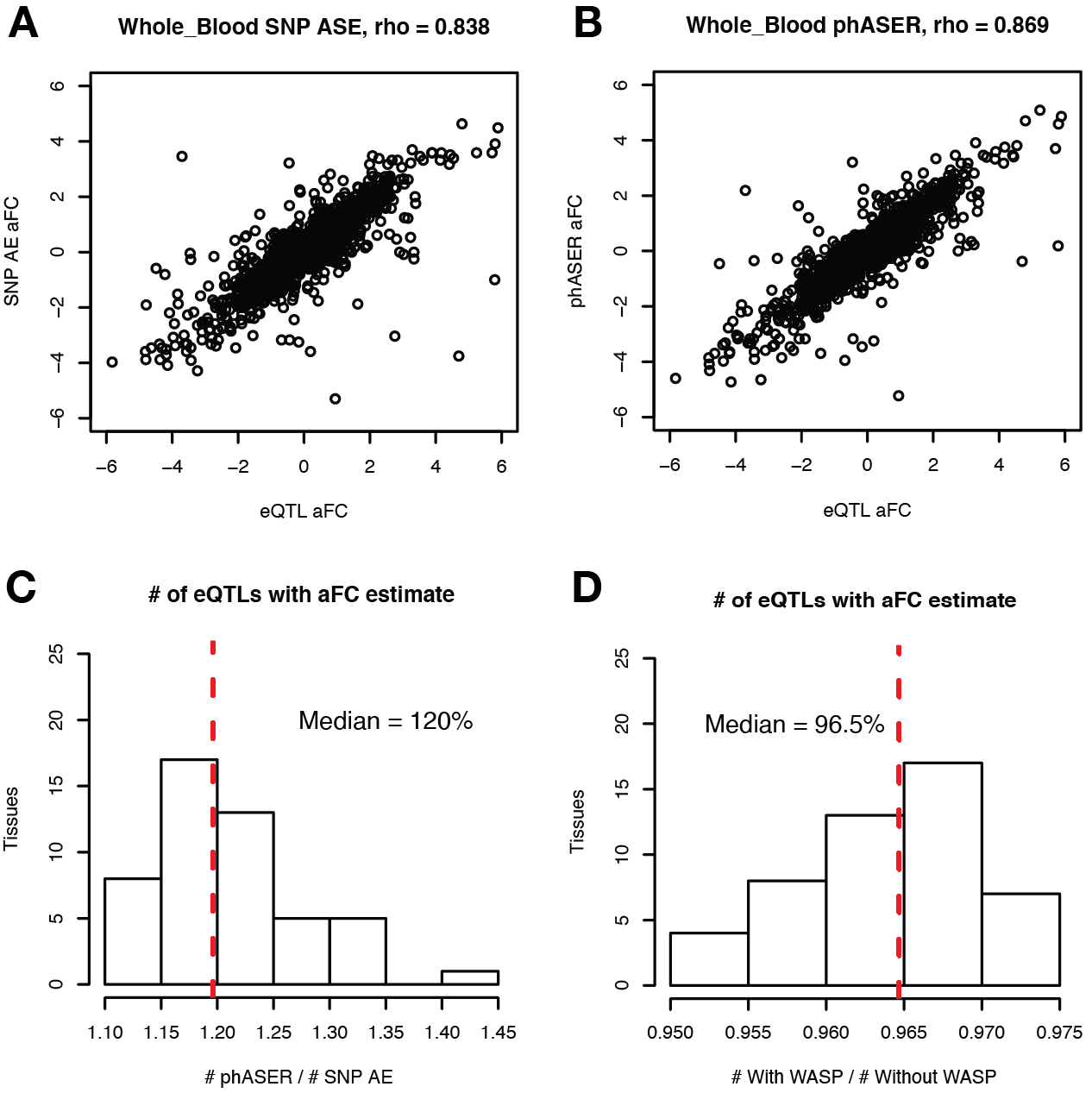


**Figure S3. Capturing *cis*-regulatory effects with phased AE data extended. A-B)** Correlation between eQTL effect size (aFC) and effect size measured using AE data from the single SNP with the highest coverage **(A)** or haplotype-level AE generated with phASER **(B)** in GTEx v8 Whole Blood. Number of eQTLs with sufficient (≥ 10 individuals) allelic expression data to estimate effect size using phASER to generate haplotype-level estimates versus using the single SNP with the highest coverage **(C)** and phASER with WASP versus without WASP filtering **(D)**.

**
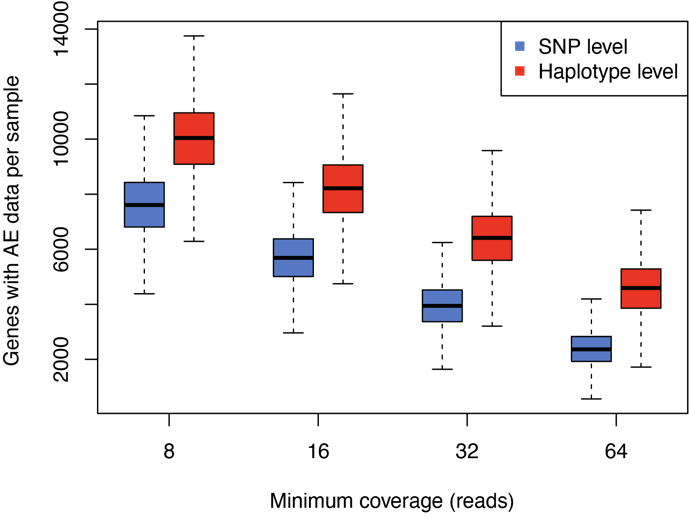
**

**Figure S4. Sample level AE data metrics.** Boxplot showing number of genes with AE data per sample across all GTEx tissues using either SNP-level (blue) or haplotype-level (red) data versus minimum read coverage. For boxplots, bottom whisker: Q1 − 1.5*interquartile range (IQR), top whisker: Q3 + 1.5*IQR, box: IQR, and center: median and outliers are hidden for ease of viewing.

**
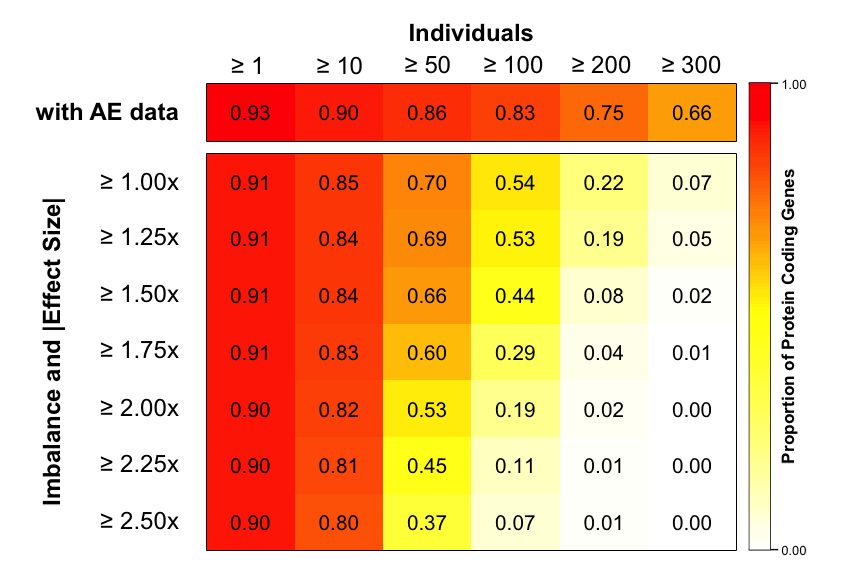
**

**Figure S5. Phased AE data at protein coding genes across tissues.** Heatmap showing the proportion of protein coding genes with AE data in at least one GTEx tissue as a function of the number of individuals with data using all AE data (top row) or only those samples with significant imbalance (binomial test, sample level FDR < 5%) and increasing minimum effect size, calculated using aFC and listed in exponentiated form (2^aFC^).
