# Supplementary material for: A vast resource of allelic expression data spanning human tissues
Source: Genome Biol. 2020 Sep 11;21:234. doi: 10.1186/s13059-020-02122-z (PMC7488534; doi:10.1186/s13059-020-02122-z)
Supplement: Supplementary file 4 — Additional file 4. Review history. [file 13059_2020_2122_MOESM4_ESM.docx]

Review History

**First round of review**

**Reviewer 1**

**Are you able to assess all statistics in the manuscript, including the appropriateness of statistical tests used?**

Yes, and I have assessed the statistics in my report.

**Comments to author:**

GTEx is a valuable resource to study the tissue specific gene expression. In this manuscript, the authors briefly described allele specific expression (ASE) analysis in GTEx v8 release. The processed data are currently available in GTEx website and can be served as an important resource to the human genetics community. I have the following comments and questions:

1. Perhaps due to the page limit, the description is quite brief. For example, what is phASER-POP? More detailed descriptions regarding your methods and data are appreciated.

2. GETx v8 has information for 54 tissues. Why have only 49 tissues been analyzed in Figure 2?

3. Both with and without using WASP filtering data have been provided. What's your recommendation for WASP filtering step?

4. What's the relationship between allele frequency and allele specific expression? Do ultra-rare variants have larger effect sizes?

5. Does the type 1 error rate is well controlled?

**Reviewer 2**

**Are you able to assess all statistics in the manuscript, including the appropriateness of statistical tests used?**

Yes, and I have assessed the statistics in my report.

**Comments to author:**

Castel et al. describe a new data resource comprising allele specific expression for the GTEx v8 samples. The novelty of the resource is its large sample size (n=838) and its coverage of multiple tissues (54 tissues). To generate the resource, the authors produced a new version of their method phASER.

The resource will clearly be useful given the literature on ASE analysis which relied on smaller sample sizes, and the paper is generally well written. However, the text and figures would benefit from revision.

Major points:

1. I find Fig 2 very difficult to interpret.

Fig 2A: I do not understand why both "total samples per tissue" and "median number of samples with sufficient ASE data" are plotted (using x-coordinate and size, respectively). For the purposes of this resource, naively I would guess that only the latter number matters, and the plot could be simplified by only plotting "Number of genes with ASE data" against "Number of samples with ASE data" (and labeling the axes as such).

I suggest using e.g., direct labels to avoid overplotting the tissue names, and using darker text to make the labels readable. I would also suggest either including the GTEx tissue group colorlegend, or not using colors.

Fig 2B: The plot shows the distribution of the number of genes which have significant allelic imbalance across the 54 tissues, for different choices of minimum sample size. This is very difficult to follow from the axis labels and legend.

It might be clearer to:

- plot the 54 values for each tissue, for each choice of minimum sample size, instead of the boxplots

- plot "Proportion of ASE genes with significant allelic imbalance" against "Minimum sample size" (and labelling the axes as such) rather than including the number of genes with sufficient ASE data in the same plot

- re-label "imbalance" as "All samples" and "imbalance-HET" as "Excluding GTEx eQTL heterozygotes"

2. Fig S5 is referenced to support the claim "strong regulatory effects...are widely present, with 47% of genes...showing such an effect". I find it difficult to interpret how the figure supports this claim: is the relevant information the cell of the heatmap marked individuals >= 50, effect size >= 1.15, proportion = 0.47?

This effect size is reported as ">2x" in the main text, but I don't understand how this follows from the aFC threshold.

Minor points:

1. The background section uses "allele specific expression", "allelic expression analysis", and "ASE analysis" inconsistently, which is confusing. I suggest re-ordering the background section to explain:

(1) ASE is imbalance in transcripts coming from the two alleles in heterozygotes (Fig 1A); (2) ASE analysis can quantify ASE from ordinary RNA-seq reads (describe how this is done at the SNP and haplotype level); (3) ASE analysis can be used to identify QTLs, imprinting, etc.

2. The data resource includes SNP-level and haplotype-level ASE, both with and without WASP correction. However, the authors recommend using WASP-corrected data for most downstream analyses, and also report that correlations with eQTLs are higher for haplotype-level data. It is unclear then why all four data sets are being made available, rather than just WASP-corrected haplotype-level ASE.

I suggest including more explanation about the circumstances under which one would prefer to use the SNP-level data over the haplotype-level data, and the uncorrected data over the WASP-corrected data.

3. Fig 1C shows the distribution of estimated Spearman correlations between effect sizes estimated using ASE, and effect sizes estimated using linear regression (eQTL mapping) across all genes for each of 54 tissues.

Similarly, Fig S4 shows the distribution of number of genes with ASE against minimum coverage over the 54 tissues.

It might be more informative to plot the 54 values (jittered) for each comparison on top of the box plots.

4. The abstract highlights the improvement to the phASER software, but it is not listed as a key contribution at the end of the Background section. I suggest including it.

**Reviewer 3**

**Are you able to assess all statistics in the manuscript, including the appropriateness of statistical tests used?**

Yes, and I have assessed the statistics in my report.

**Comments to author:**

The authors reported a large resource of allelic expression data of the GTEx v8 release, which has 54 tissues totaling >15K samples. Though the data is comprehensive (in terms of tissues, with or without WASP filter, SNP based or phASER ASE), I am not convinced of the overall scientific value of this data resource.

For a data resource to contribute to the field, it needs to 1) be broadly useful for different datasets and analyses and/or 2) provide the potential to answer important biological questions with a specific dataset (in this case, the GTEx dataset). However, this specific ASE resource does not satisfy either of the two requirements. First, it is only useful for studies using the GTEx datasets. Unlike resources such as population based whole genome sequencing datasets, which are useful for phasing/imputation of any genotype datasets, this resource is only usable in combination with other GTEx data (genotype, expression etc). Moreover, even though the authors claimed that the resource was publicly available to users without dbGAP approval , without the availability of individual level data (genotype etc), it is difficult to use this resource to generate new insights (for example on common and rare regulatory variation and imprinting, as the author mentioned in the manuscript). Therefore, the values of this resource to users without dbGaP approval is still limited. Second, the authors mentioned that the resource might be useful to study gene regulation, beyond just using the gene expression levels, for example rare regulatory variants. However, as it was written in the readme file associated with the release:" Only variants with a MAF > 1% in GTEx or that have been read-back phased with a variant with MAF > 1% were used to produce haplotype counts to ensure accurate phasing when summing counts across variants." If I understand this correctly, phased haplotype with rare variants are not included in the data resource. Therefore, to study rare regulatory variants, additional analyses of ASE is still required.

I think the availability of the GTEx ASE data is definitely great, but for this resource to be a separate paper (even a short report), more analyses need to be done to demonstrate in detail how this resource can contribute to the understanding of gene regulation in general. The paper touched briefly on this in the last paragraph of Results and Discussions (Page 5&6). The author mentioned that a mere reduction of 7.5% of the number of genes with significant imbalance in at least one sample was observed, after removing individuals that were heterozygous for any GTEx eQTL. The claim is that this demonstrated that the resource could be important to identify additional regulatory effects. I think there are many other reasons that the small amount of reduction is observed. The authors need to perform more thorough analysis for it to be convincing.

Minor comment: 1) the link (https://github.com/broadinstitute/gtex-pipeline/) that contains full settings of the alignment and haplotype phasing is broken.

2) Figure 2A is very hard to see the labels. The reason why testis has the largest number of genes with phASER data is not very clearly explained in the text.

**Authors Response**

**Point-by-point responses to the reviewers’ comments:**

Reviewer #1: GTEx is a valuable resource to study the tissue specific gene expression. In this manuscript, the authors briefly described allele specific expression (ASE) analysis in GTEx v8 release. The processed data are currently available in GTEx website and can be served as an important resource to the human genetics community. I have the following comments and questions:

1. Perhaps due to the page limit, the description is quite brief. For example, what is phASER-POP? More detailed descriptions regarding your methods and data are appreciated.

*--> Due to the tight word limit constraints of the short report format, we explained the software in detail in the Methods – Software and Availability section. However, in our revised manuscript we have added a brief description of the software to the Results and Discussion section, and point readers to the methods section for more detailed information. Furthermore, we have also include additional information on the software in the methods section.*

2. GETx v8 has information for 54 tissues. Why have only 49 tissues been analyzed in Figure 2?

*--> The GTEx v8 release only mapped eQTLs in tissues with at least 70 samples, which works out to 49 tissues. Many of the analyses in this manuscript integrate with eQTLs (including figures 1c, 2b), so only those 49 tissues where eQTLs were called can be used. For consistency, we limited the analyses in this manuscript to those 49 tissues, however, the reviewer is correct in pointing out that figure 2a does not integrate eQTLs, and thus could include all 54 tissues. As such, in our revised manuscript we include all 54 tissues in figure 2a. In addition, we clarify in the text that these analyses were performed across the 49 tissues where eQTLs were mapped.*

3. Both with and without using WASP filtering data have been provided. What's your recommendation for WASP filtering step?

--> We agree that this manuscript provides an opportunity to provide guidance on whether WASP filtering *should be used. In our initial manuscript we wrote the following: “WASP correction significantly improved correlations for both SNP- (p = 2.49e-13) and haplotype- (p = 3.55e-15) level data, so we therefore recommend using WASP-corrected data for most downstream analyses”. This point could be made more clearly, and another reviewer asked for further clarification. In our revised manuscript we have added a paragraph to the Results and Discussion section that provides further guidance on this topic. Furthermore, we performed an additional analysis (Fig. S3d) which strengthens our recommendation that WASP filtering be used for most downstream analyses.*

4. What's the relationship between allele frequency and allele specific expression? Do ultra-rare variants have larger effect sizes?

*--> This is a very interesting topic, and the reviewer is correct to point out that this resource could be used to provide some insight. In our revised manuscript we analyzed the effect of heterozygous variants in gene promoters as a function of minor allele frequency and have included an additional figure with these results (Fig. 2c). As expected, we find that rare variants tend to have larger effects on allelic imbalance than common variants, with the rarest class of variants analyzed (MAF < 0.005 in GTEx) having the strongest effects. We believe this is a nice demonstration of how the haplotype-based AE resource might be used and thank the reviewer for suggesting we include it.*

5. Does the type 1 error rate is well controlled?

*--> It’s not entirely clear which analysis the reviewer is referring to here. If they are referring to the significance of allelic imbalance, we discuss appropriate methods for this extensively in a previous review of allelic expression (Castel, 2015). Unfortunately, an extensive description about the appropriate statistical methods for allelic expression analysis is outside the scope of this short report. However, in our revised manuscript we provide an additional descriptions of allelic expression analysis in the Background section, for example, highlighting the use of binomial tests for statistical significance and pointing the reader to our previous review of the topic for more information.*

Reviewer 2:

Castel et al. describe a new data resource comprising allele specific expression for the GTEx v8 samples. The novelty of the resource is its large sample size (n=838) and its coverage of multiple tissues (54 tissues). To generate the resource, the authors produced a new version of their method phASER.

The resource will clearly be useful given the literature on ASE analysis which relied on smaller sample sizes, and the paper is generally well written. However, the text and figures would benefit from revision.

Major points:

1. I find Fig 2 very difficult to interpret.

Fig 2A: I do not understand why both "total samples per tissue" and "median number of samples with sufficient ASE data" are plotted (using x-coordinate and size, respectively). For the purposes of this resource, naively I would guess that only the latter number matters, and the plot could be simplified by only plotting "Number of genes with ASE data" against "Number of samples with ASE data" (and labeling the axes as such).

I suggest using e.g., direct labels to avoid overplotting the tissue names, and using darker text to make the labels readable. I would also suggest either including the GTEx tissue group colorlegend, or not using colors.

*--> We agree with the reviewer that Fig 2a could be presented more clearly. We have updated this figure in our revised manuscript to follow the reviewer’s excellent suggestions. First we plot "Number of genes with ASE data" against "Number of samples with ASE data" as suggested. Second, we now use standard GTEx tissue colors and symbols, and include an extended legend to the left of the figure.*

Fig 2B: The plot shows the distribution of the number of genes which have significant allelic imbalance across the 54 tissues, for different choices of minimum sample size. This is very difficult to follow from the axis labels and legend.

It might be clearer to:

- plot the 54 values for each tissue, for each choice of minimum sample size, instead of the boxplots

- plot "Proportion of ASE genes with significant allelic imbalance" against "Minimum sample size" (and labelling the axes as such) rather than including the number of genes with sufficient ASE data in the same plot

- re-label "imbalance" as "All samples" and "imbalance-HET" as "Excluding GTEx eQTL heterozygotes"

*--> We thank the reviewer for their suggestions for clarifying figure 2b. We have incorporated all of their feedback into the revised figure. In addition to the changes suggested by the reviewer, in the revised figure also show genes with allelic imbalance after removing eQTL heterozygotes as a proportion of genes with allelic imbalance using all samples, which corresponds to the value given in the text. Thanks to these changes, we believe the figure concisely conveys the reduction in genes with allelic imbalance after removing eQTL heterozygotes.*

2. Fig S5 is referenced to support the claim "strong regulatory effects...are widely present, with 47% of genes...showing such an effect". I find it difficult to interpret how the figure supports this claim: is the relevant information the cell of the heatmap marked individuals >= 50, effect size >= 1.15, proportion = 0.47?

This effect size is reported as ">2x" in the main text, but I don't understand how this follows from the aFC threshold.

*--> The reviewer is correct in identifying the relevant information in the heatmap. The confusion surrounding the magnitude results from the fact that aFC is calculated in log scale. Thus 2^1.15 = 2.22x. In our revised manuscript we changed the effect size thresholds to better represent the text (i.e. there is now a threshold for exactly 2x) and we list the effect sizes as exponentiated aFC to alleviate any confusion.*

Minor points:

1. The background section uses "allele specific expression", "allelic expression analysis", and "ASE analysis" inconsistently, which is confusing. I suggest re-ordering the background section to explain:

(1) ASE is imbalance in transcripts coming from the two alleles in heterozygotes (Fig 1A); (2) ASE analysis can quantify ASE from ordinary RNA-seq reads (describe how this is done at the SNP and haplotype level); (3) ASE analysis can be used to identify QTLs, imprinting, etc.

*--> We thank the reviewer for pointing out this inconsistency. In our revision we now consistently use allelic expression (as opposed to ASE), and better define the terms related to allelic expression analysis in the Background section.*

2. The data resource includes SNP-level and haplotype-level ASE, both with and without WASP correction. However, the authors recommend using WASP-corrected data for most downstream analyses, and also report that correlations with eQTLs are higher for haplotype-level data. It is unclear then why all four data sets are being made available, rather than just WASP-corrected haplotype-level ASE.

I suggest including more explanation about the circumstances under which one would prefer to use the SNP-level data over the haplotype-level data, and the uncorrected data over the WASP-corrected data.

*--> This important point was pointed out by another reviewer, and we agree that this manuscript provides an opportunity to provide further clarity. In our revised manuscript we have added one paragraph each to the Results and Discussion section that elaborate on recommendations for when to use SNP vs haplotype level, and with WASP vs without WASP data. Furthermore, we performed an additional analysis (Fig. S3d) which strengthens our recommendation that WASP filtering be used for most downstream analyses. We included all four data sets in the resource for the sake of completeness. There could be unforeseen circumstances under which one of the four data types would be optimal, and it is less work to provide them all upfront than to regenerate them on demand.*

3. Fig 1C shows the distribution of estimated Spearman correlations between effect sizes estimated using ASE, and effect sizes estimated using linear regression (eQTL mapping) across all genes for each of 54 tissues.

Similarly, Fig S4 shows the distribution of number of genes with ASE against minimum coverage over the 54 tissues.

It might be more informative to plot the 54 values (jittered) for each comparison on top of the box plots.

*--> We appreciate the suggestion and produced versions of the figures with jittered values on top of the box plots, however we found that the figures became extremely busy and difficult to read. As such, we have decided to keep the boxplots as is, as we believe they are the most concise and widely accepted way to present this type of data.*

4. The abstract highlights the improvement to the phASER software, but it is not listed as a key contribution at the end of the Background section. I suggest including it.

*--> In our revision we have included the improvement to the phASER software as a contribution at the end of the Background section.*

Reviewer #3: The authors reported a large resource of allelic expression data of the GTEx v8 release, which has 54 tissues totaling >15K samples. Though the data is comprehensive (in terms of tissues, with or without WASP filter, SNP based or phASER ASE), I am not convinced of the overall scientific value of this data resource.

For a data resource to contribute to the field, it needs to 1) be broadly useful for different datasets and analyses and/or 2) provide the potential to answer important biological questions with a specific dataset (in this case, the GTEx dataset). However, this specific ASE resource does not satisfy either of the two requirements. First, it is only useful for studies using the GTEx datasets. Unlike resources such as population based whole genome sequencing datasets, which are useful for phasing/imputation of any genotype datasets, this resource is only usable in combination with other GTEx data (genotype, expression etc). Moreover, even though the authors claimed that the resource was publicly available to users without dbGAP approval , without the availability of individual level data (genotype etc), it is difficult to use this resource to generate new insights (for example on common and rare regulatory variation and imprinting, as the author mentioned in the manuscript).

Therefore, the values of this resource to users without dbGaP approval is still limited.

*--> We respectfully disagree with the reviewer that this resource does not have overall scientific value or have the potential to answer important biological questions. First, as a point of clarification, this manuscript serves as the official reference and description of the entire GTEx v8 ASE data set (including SNP-level data), not just the haplotype level data, which is the datatype we are able to make publicly available.*

*--> The GTEx v6 SNP-level ASE data, which required dbGAP approval, has proven to be an invaluable resource for researchers studying gene regulation. For example, here are just a few of the numerous publications that used the GTEx v6 / v7 SNP level ASE data:*

*- Tukiainen, T., Villani, A. C., Yen, A., Rivas, M. A., Marshall, J. L., Satija, R., … MacArthur, D. G. (2017). Landscape of X chromosome inactivation across human tissues. Nature, 550(7675), 244–248.*

*- McCoy, R. C., Wakefield, J., & Akey, J. M. (2017). Impacts of Neanderthal-Introgressed Sequences on the Landscape of Human Gene Expression. Cell, 168(5), 916-927.e12.*

*- Li, X., Kim, Y., Tsang, E. K., Davis, J. R., Damani, F. N., Chiang, C., … Montgomery, S. B. (2017). The impact of rare variation on gene expression across tissues. Nature, 550(7675), 239–243.*

*- Castel, S. E., Cervera, A., Mohammadi, P., Aguet, F., Reverter, F., Wolman, A., … Lappalainen, T. (2018). Modified penetrance of coding variants by cis-regulatory variation contributes to disease risk. Nature Genetics, 50(9), 1327–1334.*

*- Mohammadi, P., Castel, S. E., Cummings, B. B., Einson, J., Sousa, C., Hoffman, P., … Lappalainen, T. (2019). Genetic regulatory variation in populations informs transcriptome analysis in rare disease. Science, 366(6463), 351–356.*

*--> As for the utility of the haplotype level ASE v8 data generated as part of this manuscript, it has already been pivotal in numerous GTEx v8 analyses, including the following:*

*- Aguet, F., Barbeira, A. N., Bonazzola, R., Brown, A., Castel, S. E., Jo, B., … Lappalainen, T. (2019). The GTEx Consortium atlas of genetic regulatory effects across human tissues. BioRxiv, 787903.*

*- Kim-Hellmuth, S., Aguet, F., Oliva, M., Muñoz-Aguirre, M., Wucher, V., Kasela, S., … Lappalainen, T. (2019). Cell type specific genetic regulation of gene expression across human tissues. BioRxiv, 806117.*

*- Ferraro, N. M., Strober, B. J., Einson, J., Li, X., Aguet, F., Barbeira, A. N., … Battle, A. (2019). Diverse transcriptomic signatures across human tissues identify functional rare genetic variation. BioRxiv, 786053.*

*- Goede, O. M. de, Ferraro, N. M., Nachun, D. C., Rao, A. S., Aguet, F., Barbeira, A. N., … Montgomery, S. B. (2019). Long non-coding RNA gene regulation and trait associations across human tissues. BioRxiv, 793091.*

*--> Finally, the haplotype level data can be useful even without genotype data. Examples of this include analysis of constraint on regulatory variation affecting gene expression (Mohammadi et al. 2019 Science) and some approaches for imprinting and X-chromosome inactivation analysis.*

*--> Therefore, as demonstrated above, we believe the GTEx v8 ASE data resource presented in this manuscript is of high overall scientific value and will have the potential to answer important biological questions. Furthermore, we believe that via this work, the more accessible and carefully documented data set will further increase future use. We also point out that other reviewers believed that the resource “serve[s] as an important resource to the human genetics community” and “will clearly be useful given the literature on ASE analysis which relied on smaller sample sizes”.*

Second, the authors mentioned that the resource might be useful to study gene regulation, beyond just using the gene expression levels, for example rare regulatory variants. However, as it was written in the readme file associated with the release:" Only variants with a MAF > 1% in GTEx or that have been read-back phased with a variant with MAF > 1% were used to produce haplotype counts to ensure accurate phasing when summing counts across variants." If I understand this correctly, phased haplotype with rare variants are not included in the data resource. Therefore, to study rare regulatory variants, additional analyses of ASE is still required.

*--> The reviewer is correct in their interpretation that read counts from expressed heterozygous variants < 1% MAF are not included in the haplotype-level phASER estimates (in the SNP-level estimates they are included). In analysis of rare regulatory variants, those with putative post-transcriptional effect, for example, stop gain, splice, or UTR variants would not be themselves included in the haplotype-level ASE data. In the case of non-transcribed variants (e.g. enhancer or promoter variants) these would not be included in the AE data regardless, because they have no RNA-seq reads overlapping them. In either circumstance, haplotype-level data captures the cumulative effect of all cis-regulatory variation, not just the effects of the variant(s) which AE is being measured at, which is an important distinction. In the Background section of our manuscript we point this out: “In practice, AE analysis uses RNA-seq reads that overlap heterozygous single nucleotide polymorphisms (SNPs), where the SNP can be used to assign the read to an allele. These heterozygous SNPs capture the cumulative effects of cis-regulatory variation acting on each allele… In some cases, these effects can be caused by the SNPs being used to measure AE themselves, for example, stop gain variants that cause nonsense-mediated decay (NMD; Rivas, 2015), but often they simply capture effects of other cis-acting variation.”. Because these SNPs capture the cumulative effects of all cis-regulatory variants, even if a specific variant is not included in the haplotype-based allelic expression measurement, the measurement will still capture the effect of the rare variant if the individual has any other heterozygous variant in the gene. If the goal is to specifically analyze rare transcribed variants with post-transcriptional effects, then the SNP-level AE data would be more straightforward, however, for all other purposes including analysis of rare regulatory variants affecting transcription, the haplotype-level data is better to use. In our revised manuscript we have added a paragraph to the Results and Discussion section that provides guidance on when the haplotype-level versus SNP-level AE should be used.*

I think the availability of the GTEx ASE data is definitely great, but for this resource to be a separate paper (even a short report), more analyses need to be done to demonstrate in detail how this resource can contribute to the understanding of gene regulation in general. The paper touched briefly on this in the last paragraph of Results and Discussions (Page 5&6). The author mentioned that a mere reduction of 7.5% of the number of genes with significant imbalance in at least one sample was observed, after removing individuals that were heterozygous for any GTEx eQTL. The claim is that this demonstrated that the resource could be important to identify additional regulatory effects. I think there are many other reasons that the small amount of reduction is observed. The authors need to perform more thorough analysis for it to be convincing.

*--> As mentioned in a previous response to this reviewer, the allelic expression data set has already found many uses in GTEx v8 companion papers, demonstrating its widespread use to understand gene regulation. To highlight the broad usefulness of the resource for understanding gene regulation in our revision we have expanded on the Conclusion section by highlighting a multitude of studies that have made use of both the v8 resource as well as previous versions. In addition, in our revised manuscript we have performed an additional analysis that demonstrates how the resource can be used to study the effects of rare regulatory variation as requested by the reviewer. We analyzed the effect of heterozygous variants in gene promoters as a function of minor allele frequency and have included an additional figure with these results (Fig. 2c). As expected, we find that rare variants tend to have larger effects on allelic imbalance than common variants, with the rarest class of variants analyzed (MAF < 0.005 in GTEx) having the strongest effects. This clearly demonstrates that haplotype-based AE data is useful for identifying rare regulatory effects. Further analysis is outside the scope of this short resource paper but is an interesting topic that can be pursued in future.*

Minor comment: 1) the link (https://urldefense.proofpoint.com/v2/url?u=https-3A__github.com_broadinstitute_gtex-2Dpipeline_&d=DwIGaQ&c=C9X8xNkG_lwP_-eFHTGejw&r=WS5i10iDRUEF_TPa-u27fYZ2B_qhINc8z5H45alE4yA&m=ACuUPW_RTWKG2uoe5oUV66p8LcUuxNl_efQDsAqCENE&s=Akk_IVZpb2lww3acF4U6ej_3Sir3nyXF6b-45QD4D_Q&e= ) that contains full settings of the alignment and haplotype phasing is broken.

*--> The text of the link “https://github.com/broadinstitute/gtex-pipeline/” does work, however it appears that embedded link got corrupted. In our revision, we have removed the embedded link, so we expect that readers will copy paste the correct link into their web browser.*

2) Figure 2A is very hard to see the labels. The reason why testis has the largest number of genes with phASER data is not very clearly explained in the text.

*--> In our revised manuscript we have improved the readability and interpretability of figure 2A based on suggestions from other reviewers. Furthermore, we have included a new supplementary table (Table S1) with the underlying data from figure 2a, which includes sample size, number of expressed genes, number of genes with phASER data, and the median number of samples with phaser data per gene. Finally, we have expanded upon our description of the number of genes with phASER data by performing correlations with number of genes expressed and sample size and explaining why the testis have the most genes with phaser data.*

**Second round of review**

**Reviewer 1**

The manuscript is well written and much more clear now. The authors have addressed all of my comments satisfactory. I really appreciate the authors make haplotype-level AE data publicly available.

I just have one suggestion. The authors may consider explaining aFC per sample and at the population level more explicitly to make it clear.

**Reviewer 3**

1. I agree and have always agreed that AE data is of crucial importance and is the key to many new biological insights. However, maybe I wasn't clear in my previous comment, my doubt over the value of the dataset is that, 1) without the availability other access-restricted GTEx data, including genotypes, expressions and covariates etc, the GTEx AE data alone is not going to help us gain those insights. I had this doubt because the manuscript seemed to be written in a way that emphasized the importance of the "public available" haplotype AE dataset. I agree that the haplotype AE is more suitable for downstream analyses in many circumstances, in comparison to SNP-level data. However, I think the author should tone down the "public available" part (eg., from the abstract, and conclusion), because as I mentioned, the public available haplotype dataset alone could hardly be used to get to biological insights. 2) the GTEx v8 AE dataset could only benefit analyses using the GTEx data. Though in my opinion, an impactful resource should benefit a broader scientific community. Many new large scale RNAseq datasets are being generated in labs worldwide nowadays, and this resource would not contributed to the analyses of these new data. I don't doubt the pivotal role of the GTEx, but I have to admit that I still doubt the necessity that the AE data become a standalone resource. I understand this is a difference in opinion, and it is not the authors responsibility to justify this, so I don't expect the authors to respond to this.

2. I am satisfied with the authors' response.

3. I am largely satisfied with the authors' response. The analysis showing larger effect sizes of rarer variants is nice and expected, but it doesn't mean that the imbalance in the majority (at least 60%) of the genes after removing any GTEx heterogenous eQTLs is due to rare variants, but I agree the detailed analyses is out of scope for this short report. However, I would suggest an edit to the text: from "..demonstrating the potential of this resource to identify additional, likely rare regulatory effects…" to "..demonstrating the potential of this resource to identify additional regulatory effects, including rare regulatory effects".

4. I am satisfied with the authors' response on the minor points.

**Authors Response**

**Point-by-point responses to the reviewers’ comments:**

Response to reviewer 1:

I just have one suggestion. The authors may consider explaining aFC per sample and at the population level more explicitly to make it clear.

*> Thank you for the suggestion. We have expanded the end of the first paragraph of the Results and Discussions section to better explain sample versus population level aFC interpretations and usage.*

Response to reviewer 3:

I am largely satisfied with the authors' response. The analysis showing larger effect sizes of rarer variants is nice and expected, but it doesn't mean that the imbalance in the majority (at least 60%) of the genes after removing any GTEx heterogenous eQTLs is due to rare variants, but I agree the detailed analyses is out of scope for this short report. However, I would suggest an edit to the text: from "..demonstrating the potential of this resource to identify additional, likely rare regulatory effects…" to "..demonstrating the potential of this resource to identify additional regulatory effects, including rare regulatory effects".

*> We have changed the text as requested by the reviewer.*
